# The High Expression of Legumain in Canine Neoplasms: A Retrospective Analysis of 100 Cases

**DOI:** 10.3390/ani12040504

**Published:** 2022-02-17

**Authors:** Chiao-Hsu Ke, Ka-Mei Sio, Shang-Lin Wang, Ying Kuo, Wei-Hsiang Huang, Chen-Si Lin

**Affiliations:** 1 Department of Veterinary Medicine, School of Veterinary Medicine, National Taiwan University, Taipei 10617, Taiwan; f08629002@ntu.edu.tw (C.-H.K.); r10629010@ntu.edu.tw (K.-M.S.); b06609002@ntu.edu.tw (Y.K.); 2 Graduate Institute of Veterinary Clinical Science, School of Veterinary Medicine, National Taiwan University, Taipei 10617, Taiwan; shanglinwang@ntu.edu.tw; 3 Animal Cancer Center, College of Bioresources and Agriculture, National Taiwan University, Taipei 10617, Taiwan; 4 National Taiwan University Veterinary Hospital, College of Bioresources and Agriculture, National Taiwan University, Taipei 10672, Taiwan; 5 Graduate Institute of Molecular and Comparative Pathobiology, School of Veterinary Medicine, National Taiwan University, Taipei 10617, Taiwan

**Keywords:** legumain, comparative oncology, immunohistochemistry, tumor antigen, dog

## Abstract

**Simple Summary:**

Cancer is the leading cause of death in humans and is one of the most common canine diseases. The similarities in pathological features and tumor behaviors between spontaneous canine tumors and their human counterparts make dogs ideal models for comparative cancer research. Legumain is a novel asparaginyl endopeptidase that is overexpressed in numerous types of human tumors. Furthermore, legumain-targeted cancer therapy has been proposed, and the treatment efficacy is well-tolerated. Previous studies have shown that legumain regulates extracellular matrix degradation and triggers the invasion and the metastasis of tumors. However, in dogs, the role of legumain in the progression of tumors remains largely unknown, and few investigations have described the expression levels of this protein in canine tumors. The present study was carried out to evaluate whether legumain is expressed in ten different types of canine neoplasms. We found that heightened signals of legumain were expressed in all canine tumor samples in the study, and, notably, the non-mesenchymal types of tumors harbored relatively high expression levels. This study is the first to describe the legumain distribution pattern in a series of canine tumors. Though further investigation is needed, the current study has provided large-scale pan-screening data on legumain as a potential biomarker, or a therapeutic target, in veterinary oncology.

**Abstract:**

Legumain, a novel asparaginyl endopeptidase, has been observed to be overexpressed in several types of human solid tumors. Elevated levels of legumain are found in human cancers, and this oncoprotein may facilitate tumor invasion and metastasis when overexpressed. These findings suggest that legumain plays a malignant role in cancer biology. However, currently, no publications have identified the role of legumain in the development of canine cancers. The present study first compared the expression patterns of legumain in paraffin-embedded canine tumor tissues, with those of normal tissues, by immunohistochemistry. A total of 100 canine tumor samples, including mast cell tumors, soft tissue sarcoma, hemangiosarcoma, lymphoma, mammary gland carcinoma, hepatoid gland tumor, squamous cell carcinoma, trichoblastoma, and melanoma were evaluated. Compared with the normal tissues, all tumor samples displayed high intensities of legumain expression. Mesenchymal-type tumors displayed immunoreactivity for legumain, with an average expression of 40.07% ± 1.70%, which was significantly lower than those of epithelial tumors and other types of tumors, which had median expressions of 49.12% ± 1.75% and 47.35% ± 2.71%, respectively (*p* < 0.05). These findings indicate that legumain has a high potential to be a candidate for distinguishing tumors from normal tissues. Although further studies on a larger number of cases are necessary to clarify the clinical application of legumain, the overexpression patterns of legumain in canine tumor tissues are reported, for the first time, in this study.

## 1. Introduction

Legumain is a lysosomal protease and a cysteine endopeptidase of asparaginyl endopeptidase (AEP), which displays a high specificity for the hydrolysis of the C13 family [1]. Legumain is overexpressed in several types of tumors and exhibits a high level of mRNA expression in tumors [2]. Limited legumain expression can be detected in normal tissues [3], whereas numerous human cancers, such as ovarian cancer [4], colorectal cancer [5], prostate cancers [6], gastric carcinoma [7], and breast cancers [3] are known to express elevated levels of legumain. Furthermore, its over-expression has been found to be associated with the tumor invasion and metastasis [2], which may result from the characteristics and functions of this protein. Legumain is not only located in the intracellular regions of tumors; it is also highly expressed on the surface of tumors and tumor-associated endothelial cells, where it is colocalized with integrins [8]. The heightened expression of legumain activates gelatinase A zymogen, a vital regulator of extracellular matrix degradation, and, thereby, makes the tumors’ more malignant counterparts [9,10]. Therefore, an increased level of legumain on the cell surface could trigger the invasion and metastasis of tumors [10]. The malignant behaviors of tumors are also proposed to be related to the proteolytic function of legumain, which can activate other protease zymogens. Some of these activated proteases are linked to angiogenesis and tumor cell proliferation. Legumain-induced protease cascades are highly correlated with coagulation, apoptosis, complement cascades, and other tumor-promoting biological pathways [2,5]. Thus, legumain has been regarded as a candidate tumor antigen that is overexpressed in several types of cancers and facilitates tumor malignancies.

Companion dogs are ideal models for comparative oncology because they live relatively close to, and share highly similar tumor behaviors with, humans. In investigations of tumor biology, a canine model can mimic the actual immune status of humans because they are outbred, compared with other laboratory animals, providing a diverse gene background similar to that in human populations [11]. In dogs and humans, cancer initiation and development are influenced by several factors, including age, nutrition, immune status, and environmental stimulation [12,13,14]. Furthermore, a diverse range of cancers can be found in dogs and humans, and the histopathological features are highly similar [11]. In addition to these properties, the most important property is that the deciphering of the canine genome shares high similarities with that of the human genome [15,16], and the genetic molecular alterations that drive cancers in dogs and humans are highly analogous. The oncogenes and tumor suppressors that have been identified in human cancers, such as the mutation of *KIT* [17] in mast cell tumors, and the alteration of p53 in mammary gland tumors [18,19], also contribute to canine neoplasms. Numerous tumor-related genes and/or antigens were initially identified in humans and were later found to have similar tumor promotion roles in canine cancers.

Legumain, a tumor-associated antigen, has been widely investigated in human oncology to determine its malignant roles and its potential tumor-promoting mechanisms; however, few studies have considered the expression of legumain in tumor-bearing dogs. This study aimed to evaluate the expression of legumain in tumor-bearing dogs, because tumor antigens are useful tools in cancer diagnoses, monitoring, prognoses, and even as therapy targets. In addition, whether the anti-legumain polyclonal antibody can cross-react with dogs needs to be considered. Therefore, the main purpose of this study was to compare the legumain expression in various canine tumors with that in normal tissues. Second, this study also assessed the utility of an anti-legumain polyclonal antibody that can specifically recognize canine tumors.

## 2. Materials and Methods

### 2.1. Selection of Samples

All the tumor samples were collected for diagnostic purposes at the National Taiwan University Veterinary Hospital (NTUVH), Taipei, Taiwan, and the investigators had no influence on the execution of any clinical procedures. The owners provided informed consent to use the clinical data and the excised tumors for teaching and research purposes. A total of 100 tumor samples obtained during surgery between 2016 and 2021 were selected, including the mast cell tumor (MCT, 20 cases), soft tissue sarcoma (STS, 10 cases), hemangiosarcoma (HAS, 10 cases), lymphoma (10 cases), mammary gland tumor (MGT, all were carcinomas, 10 cases), hepatoid gland tumor (HGT, 10 cases), squamous cell carcinoma (SCC, 10 cases), trichoblastoma (10 cases), oral melanoma (OM, 5 cases), and skin melanoma (SM, 5 cases). According to their origin, these tumors were further allocated into three groups: mesenchymal-type tumors (MCT, lymphoma, HAS, and STS), epithelial-type tumors (SCC, MGT, HGT, and trichoblastoma), and melanomas (OM and SM). Mesenchymal cells originate from the mesoderm [20], whereas melanomas are neoplasms of neuroectodermal origin [21]. Therefore, melanomas were excluded from the mesenchymal tumor group. Samples of the control canine bodies were immediately collected upon body donation. No causes of death in the control group were related to tumors. The owners also signed informed consent to use the clinical data and the samples for research purposes. All tissues were fixed in 10% neutral formalin and were paraffin-embedded. All the tumor samples were histologically diagnosed by two veterinary pathologists of the Graduate Institute of Molecular and Comparative Pathobiology, National Taiwan University, Taipei, Taiwan. Histopathology slides and formalin-fixed, paraffin-embedded (FFPE) tissue sections were retrieved, and all slides were further reviewed by the same veterinary pathologist (Wei-Hsiang Huang) to increase the validity of diagnoses in this retrospective study. This study was approved by the Institutional Animal Care and Use Committee, National Taiwan University (NTU-105-EL-00007).

### 2.2. Immunochemistry

To evaluate the legumain expression, blocks were sectioned at 4 µm and deparaffinized in non-xylene (Muto Pure Chemicals Co., Ltd., Tokyo, Japan), and then they underwent antigen retrieval in 1× Trilogy solution (pH = 7.0) (Sierra College Boulevard, Rocklin, CA, USA, diluted with deionized water) at 114–121 °C for 5 min in a pressure cooker (Montage Opus™ (Diagnostic BioSystems, CA, USA)). The Novolink Polymer Detection System (Leica Biosystems, Wetzlar, Germany) was applied for the immunohistochemistry. To block non-specific endogenous binding, sections were treated with a peroxidase block solution (Novolink™ Polymer Detection System, Newcastle, UK) for 5 min and were then incubated with a protein block solution (Novolink™ Polymer Detection System, Newcastle, UK) for 5 min. Slides were incubated with a primary antibody, the rabbit anti-legumain polyclonal antibody, diluted by 1:200 (LSBio, catalog no. LS-B15611, Seattle, USA) at room temperature for 1 h, followed by a Post Primary solution at room temperature for 30 min (Novolink™ Polymer Detection System, Newcastle, UK), and then the Novolink Polymer at room temperature for 30 min. An AEC (3-Amino-9-ethylcarbazole) substrate was used to develop the staining reaction, and the nuclei were stained with hematoxylin. The sections were stained with an anti-legumain antibody, and in the negative controls, sections were incubated with the normal serum (diluents) instead of the primary antibody.

### 2.3. Evaluation of Immunochemistry

To evaluate the legumain expression in all tissue sections, the estimated percentages of the immunopositivity of each slide were independently analyzed in five random microscopic fields at 40× objective magnification (0.1 mm^2^) and were then separately analyzed by two veterinary pathologists, W.-H.H. and C.-S.L. (NTUVH), who were blinded to the experimental history. To avoid artifacts, sections in the areas with necrosis, and areas in the margins of the tissues, were not considered.

### 2.4. Cell Culture

Canine diffuse large B-cell lymphoma (CLBL-1) [22] and T-cell lymphoma (UL-1) [23] cell lines were maintained in RPMI-1640 (Thermo Fisher Scientific, catalog no. 11875093) supplemented with 10% fetal bovine serum (Thermo Fisher Scientific, catalog no. 10100147) and 1% antibiotic–antimycotic (Simply, catalog no. CC501-0100). Canine mammary gland tumor cell lines, CMT-1 [24] and MPG, and canine melanoma cell lines, KMec [25] and M5 [26], were cultured in Dulbecco’s Modified Eagle Medium (DMEM, HyClone, catalog no. SH30243.02) containing 10% fetal bovine serum and 1% antibiotic–antimycotic. All the cell lines were cultured in a humidified incubator with 5% CO_2_ at 37 °C.

### 2.5. Protein Extraction and Western Blot Analysis

To validate the specificity and cross-reactivity of this polyclonal antibody with canine tumors, a Western blot analysis was performed using different tumor cell lines. All canine tumor cell lines were harvested, washed three times with PBS, and were lysed in a RIPA lysis buffer (Sigma-Aldrich, St. Louis, MO, USA, catalog no. R0278). After 30 min of incubation at 4 °C, the proteins were collected by centrifugation at 12,000× *g* for 20 min. The concentration of proteins in each sample was quantified by a BCA protein assay (Bio-Rad Laboratories, Hercules, CA, USA). Then, the protein samples (20 µg) were separated into 10% acrylamide SDS-PAGE gel and were transferred to a PVDF membrane. The membrane was blocked with a blocking buffer (5% skim milk in TBS) for 1 h at room temperature and then incubated with an anti-legumain antibody or anti-alpha tubulin (Abcam, catalog no. ab7291) at a 1:1000 or 1:5000 dilution in the blocking buffer overnight at 4 °C. The membrane was then washed with TBST (0.05% Tween-20 in TBS) for 30 min and incubated with a horseradish peroxidase (HRP)-conjugated anti-rabbit (Invitrogen, catalog no. 65-6120) or anti-mouse (Invitrogen, catalog no. 62-6520) secondary antibody, diluted to 1:5000 in 1% skim milk for 1 h at room temperature. After washing, the signal was developed by chemiluminescence using the ECL prime kit (Bio-Rad Laboratories, Hercules, CA, USA).

### 2.6. Statistical Analysis

The data were described as the mean ± standard error of the mean (SEM). Statistical analyses were performed in GraphPad Prism version 8. To determine significant differences, the Kruskal–Wallis test was utilized. Differences were considered statistically significant at a *p*-value of less than 0.05.

## 3. Results

### 3.1. Specificity of the Anti-Legumain Polyclonal Antibody

**Figure 1:**
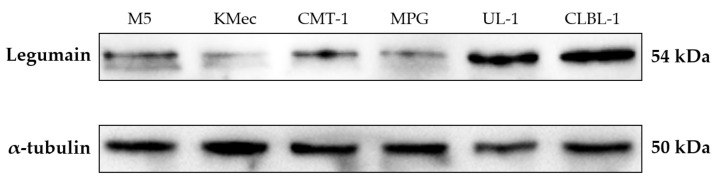
Western blot analysis of the specificity of anti-legumain polyclonal antibody in different canine cancer cell lines. Representative canine cell lines, including canine melanoma (M5 and KMec), mammary gland tumor (CMT-1 and MPG), and lymphoma (UL-1 and CLBL-1), were selected. Protein lysate of M5, KMec, CMT-1, MPG, UL-1, and CLBL-1 cell lines are shown. All the samples presented a dominant band near 54 kDa, indicating that this antibody can identify the corresponding proteins in canine species.

The results of Western blot analysis are shown in Figure 1. The anti-legumain polyclonal antibody recognized a dominant band near 54 kDa (NCBI reference sequence: XP_038528856.1) in the canine tumor cell lysates, indicating that this polyclonal antibody can identify the correspondent proteins in the canine species.

### 3.2. Low Expression Levels of Legumain Were Identified in Normal Tissues

**Figure 2:**
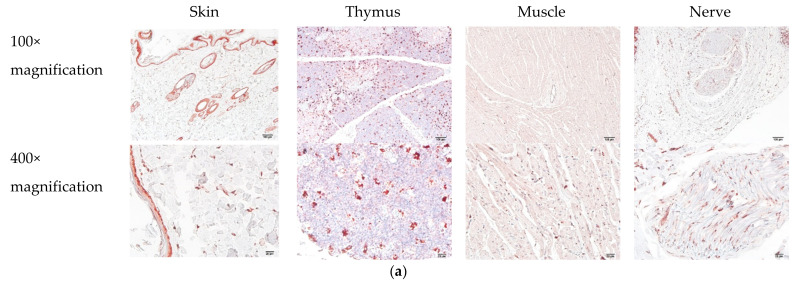

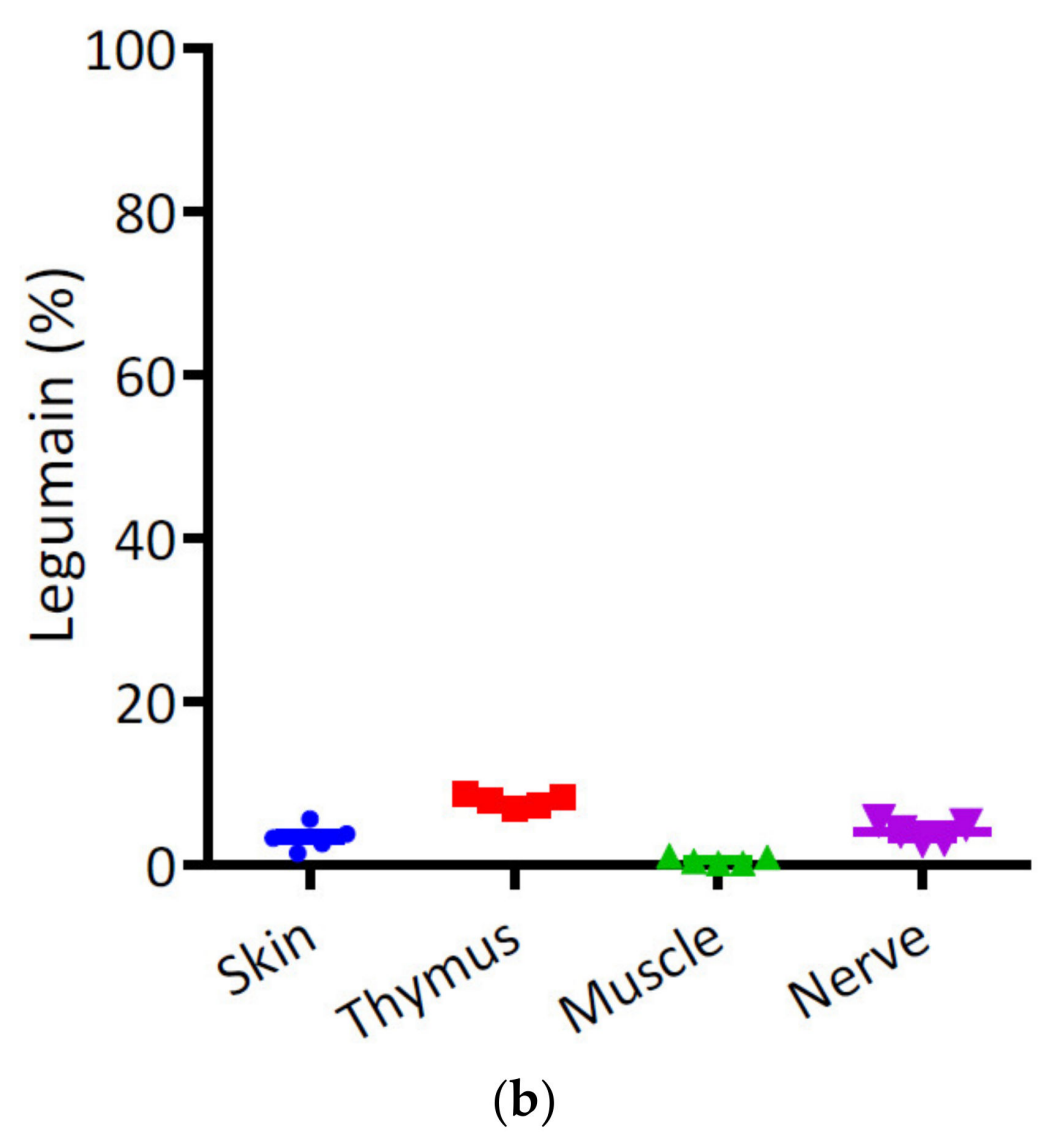
Expression patterns of legumain in canine normal tissues. Relatively low expression levels of legumain in these tissues, including (**a**) representative samples of the skin, thymus, muscle, and nerve. (**b**) Statistical analysis of legumain expression among different tissues. All data are expressed as mean ± SEM (*n* = 5).

To probe the primary antibody that would identify the normal tissues in dogs, we examined IHC in normal tissues as the control groups. The expression levels were shown as relatively negative signals of the whole figures. Figure 2a shows that there was no significant signal of legumain in the stroma of normal tissues, including the skin (Figure 2b, 3.44% ± 0.68%), thymus (Figure 2b, 7.88% ± 0.31%), muscle (Figure 2b, 0.67% ± 0.19%), and nerve (Figure 2b, 4.14% ± 0.49%) tissues. Of note, in the epidermis and in hair follicles, histiocytes demonstrated moderate-to-strong cytoplasmic legumain positivity, but the muscles, fibroblasts, and lymphocytes showed weak-to-moderated cytoplasmic immune expression.

### 3.3. High Expression of Legumain in Ten Types of Canine Neoplasms

**Figure 3:**
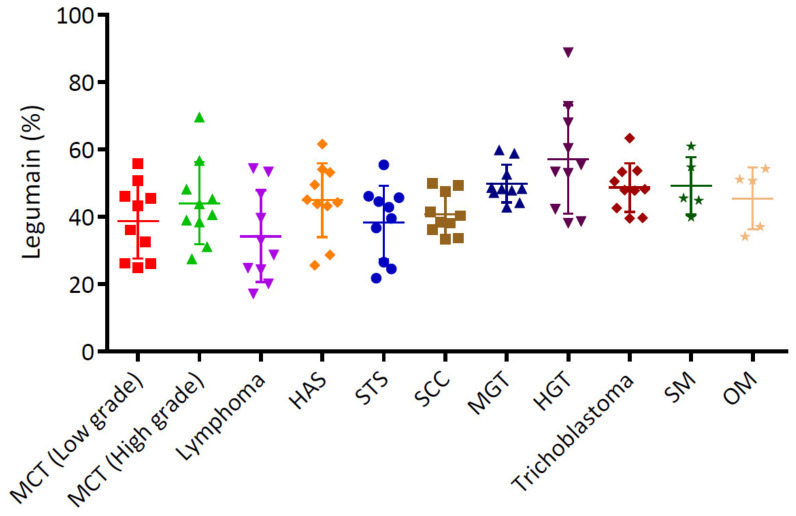
Statistical analysis of legumain expression in different types of canine tumors. Significantly elevated patterns were found in these tumors. Data are shown as mean ± SEM (*n* = 5–10). MCT, mast cell tumor; HAS, hemangiosarcoma; STS, soft tissue sarcoma; SCC, squamous cell carcinoma; MGT, mammary gland tumor (carcinoma); HGT, hepatoid gland tumor; SM, skin melanoma; OM, oral melanoma.

**Figure 4:**
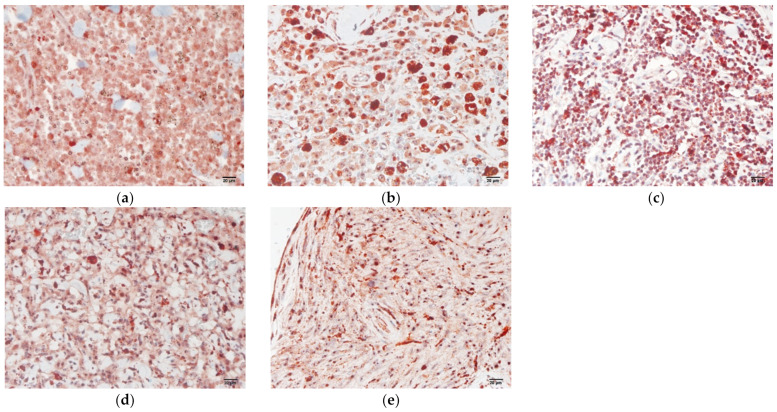
Immunochemistry staining of legumain in mesenchymal tumors. Overexpression of legumain was found in (**a**) low-grade MCT; (**b**) high-grade MCT; (**c**) lymphoma. Increased expression of legumain was found in the (**d**) HAS and (**e**) STS. Representative samples are shown and five high power fields (40× objective magnification) per tumor (*n* = 5–10) were randomly selected and scored. The bar represents 20 µm. MCT, mast cell tumor; HAS, hemangiosarcoma; STS, soft tissue sarcoma.

**Figure 5:**
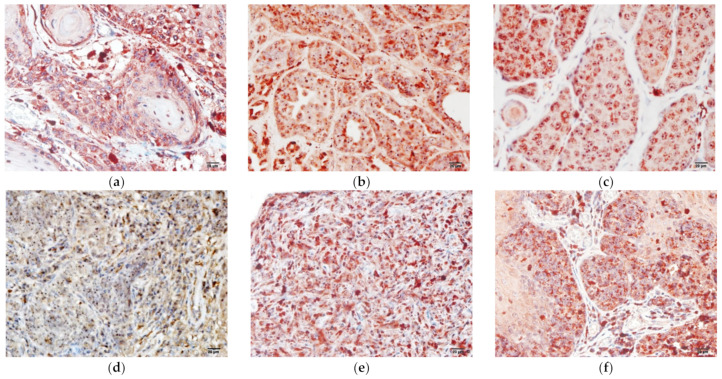
Legumain signal determined by immunochemistry. Elevated levels of legumain were found in non-mesenchymal tumors, including (**a**) squamous cell carcinoma; (**b**) mammary gland carcinoma; (**c**) hepatoid gland tumor; and (**d**) trichoblastoma. (**e**) Oral melanoma and (**f**) skin melanoma displayed heightened legumain expression. Representative samples are shown and five high power fields (40× objective magnification) per tumor sample (*n* = 5–10) were randomly selected and scored. The bar represents 20 µm.

The statistical analysis revealed that the expression percentages of legumain were significantly higher in all tumors (Figure 3) than in the normal tissues (Figure 2). Based on the source of tumor differentiation, we further divided these tumors into three groups: mesenchymal tumors (MCT, lymphoma, HAS, and STS), epithelial tumors (SCC, MGT, HGT, and trichoblastoma), and melanomas. The statistical analysis is summarized in Figure 3, and the representative figures of the tumors are separately provided in Figure 4 and Figure 5. Low-grade (Figure 4a) and high-grade MCTs (Figure 4b) expressed positive signals of 38.73% ± 3.51% and 44.05% ± 3.86%, respectively, without a significant difference (*p* > 0.05). In lymphoma cases, legumain expression was 34.23% ± 4.31% (Figure 4c). Legumain expression was observed in the cytoplasm of these round cell tumors (MCT and lymphoma). The low-grade MCTs exhibited majorly diffuse cytoplasmic patterns but occasionally exhibited vesicular patterns. In contrast, the high-grade MCTs and the lymphomas exhibited majorly vesicular and diffuse patterns. Notably, numerous neoplastic cells of the high-grade MCTs and lymphoma had occasionally moderate nuclear staining. HAS (Figure 4d), and STS (Figure 4e) had positive signals of 44.95% ± 3.49% and 38.38% ± 3.46%, respectively. The immunopositivity of legumain was also located in the diffuse cytoplasm, while STS exhibited weak vesicular positivity. The immunopositivity of legumain was also located in the diffuse cytoplasm, while STS exhibited occasionally vesicular positivity. SCC (Figure 5a) and MGT (Figure 5b) had legumain expression signals of 40.83% ± 1.95% and 49.89% ± 1.78%, respectively. Among all the samples, the HGT (Figure 5c) had the highest expression level of legumain at 57.09% ± 5.10% in the tumor sections. Trichoblastoma (Figure 5d) had a legumain signal of 48.68% ± 2.28%. SCC and MGT exhibited vesicular and diffuse patterns. The HGT and trichoblastoma exhibited majorly diffuse and tiny dotted, but occasionally vesicular, patterns. The stomal cells of these tumors exhibited almost negative signals. Oral melanoma (Figure 5e, 49.21% ± 3.80%) and skin melanoma (Figure 5f, 45.49% ± 4.11%) had no significant differences in legumain expressions. Both these melanomas exhibited strongly vesicular cytoplasmic positive signals. In summary, legumain is overexpressed in tumors; however, no significant differences in the expression of legumain were found among the ten types of tumors. To probe the possibility of different patterns among mesenchymal-type cancers, epithelial-type cancers, and melanomas, we further analyzed the expression levels among these groups.

### 3.4. Heightened Expression of Legumain in Non-Mesenchymal Tumors

**Figure 6:**
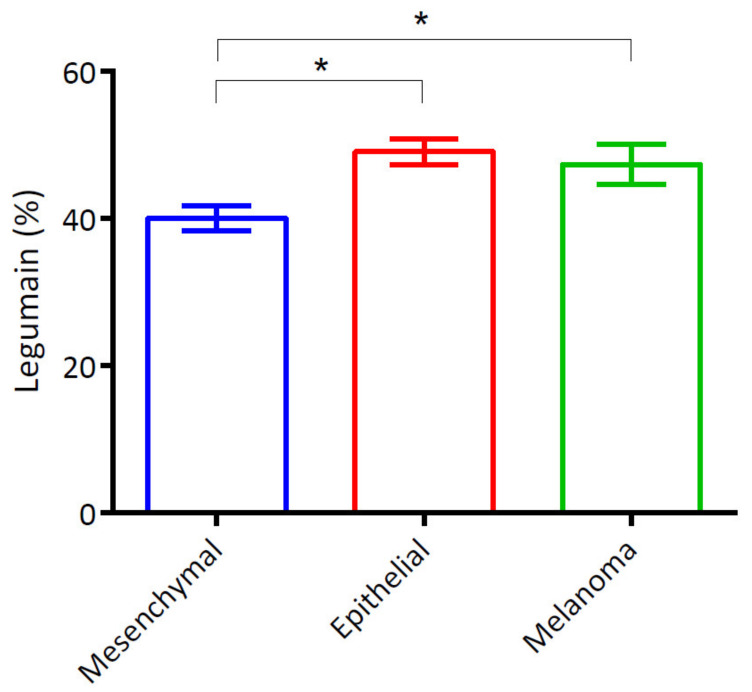
Statistical analysis of legumain expression among three tumor types. Legumain expressions in melanoma and epithelial tumors were higher than those in mesenchymal tumors. All data are expressed as mean ± SEM. Statistical significance was calculated with the Kruskal–Wallis test. * *p* < 0.05.

A total of 50 cases, distributed in four types of tumors (MCT, lymphoma, HAS, and STS), belonged to the mesenchymal-type tumor, with an average legumain expression of 40.07% ± 1.70%. Epithelial-type tumors, including SCC, MGT, HGT, and trichoblastoma, had significantly higher legumain expressions (on average, 49.12%; SEM, 1.75%) compared to mesenchymal-type tumors (Figure 6, *p* = 0.0177). The average expression of 47.35% ± 2.71% in melanomas was significantly higher than that in mesenchymal-type tumors (Figure 6, *p* = 0.0284). These results indicate that legumain is overexpressed in tumors, especially in non-mesenchymal-type tumors.

## 4. Discussion

The discovery of ideal tumor antigens that can monitor and/or predict the progression of tumors remains an important issue in human and veterinary medicine. Many tumor antigens with a prognostic value have been widely used in human medicine due to the urgent need for precision medicine. Likewise, in veterinary medicine, the identification of reliable tumor markers has gradually increased. The malignant role of legumain has been proposed; however, few publications to date have considered the malignancy of legumain in veterinary medicine. Here, we reported, for the first time, that legumain, a widely-used cancer biomarker in human oncology, is also significantly expressed in ten types of canine neoplasms.

To the authors’ best knowledge, this is the first study to evaluate legumain expression in ten types of canine tumors. Thus far, little is known about the biological functions and processes involving legumain in tumor development in dogs. However, in human oncology, the overexpression, or the potent malignant mechanisms, of legumain have been observed in ovarian cancer [4], colorectal cancer [5], prostate cancers [6], gastric carcinoma [7], and breast cancers [3]. Even though the direct correlation between tumor progression and legumain has not been widely described, the presence of cysteine endopeptidases, such as cathepsins B and L, which are activated by the legumain-mediated hydrolysis of asparaginyl bonds, may contribute to tumor malignancy [8]. This legumain-induced hydrolysis also leads to the activation of protease zymogens, which are highly correlated with angiogenesis and other tumor-facilitating functions [2,5]. Furthermore, legumain regulates the remodeling of the extracellular matrix through the initiation of the gelatinase A zymogen which, thereby, promotes tumor progression [9,10]. These underlying mechanisms may explain the overexpression of legumain found in the canine tumor tissues in the current study. We speculated that, first, through the overexpression of legumain, tumors possibly acquire the ability to display tumor-promoting phenotypes (through the extracellular matrix remodeling) and, thus, the legumain-mediated hydrolysis results in the malignant growth of tumors, especially in epithelial-type tumors.

In human medicine, Gawenda et al., reported three staining patterns of legumain, namely, diffuse positivity, tiny dots, and vesicles, in the cytoplasm, and found that breast tumors with legumain, expressed as a vesicular positivity, were correlated with a worse prognosis [3]. Ohno et al., also reported similar findings for prostate cancers, which showed that the vesicular staining patterns of legumain in these tumors had a positive correlation with tumor invasion and aggressiveness [6]. Similarly, in gastric tumors, the vesicular positivity of legumain was found in the cytoplasm of these neoplasms, whereas diffusely positive staining patterns were displayed in the normal mucosa tissues [7]. Taken together, these results indicated that the vesicular positivity of legumain in the cytoplasm could serve as a potential predictor for tumor malignancy. In the current study, we found that, in all the canine tumors, legumain was expressed in the cytoplasm. Interestingly, epithelial-type tumors and melanomas displayed a predominant vesicular positivity of legumain. Vesicles were scattered in the cytoplasm. In contrast, the mesenchymal-type tumors expressed predominant diffuse cytoplasmatic patterns, especially in the mast cell tumors, lymphoma, and hemangiosarcoma. Previous studies focused on the expression patterns in one specific type of tumor using a large sample size. In this pilot study in veterinary medicine, however, we failed to find a correlation between the tumor malignancy and the expression patterns of legumain in one type of canine tumor. Further investigations in veterinary medicine, with larger sample sizes, are needed to clarify whether the expression pattern of immunoactivity located in the cytoplasm is also correlated with the prognosis, as widely proposed in human medicine.

Our study shows that legumain is overexpressed in non-mesenchymal-type tumors. The underlying mechanisms by which these tumors displayed high levels of legumain remain unclear, even in human medicine. One previous study reported that the expression of legumain in tumor epithelial cells is associated with the potential epithelial cell-intrinsic role of early-stage tumors in the extracellular matrix degradation that facilitates tumor progression [27]. Furthermore, legumain could induce endopeptidase activity [8,28], which is most intense in the epithelial region [29]. Lastly, the expression patterns of legumain in the cytoplasm are correlated with tumor malignancy [3,6,7]. The majority of non-mesenchymal-type tumors exhibited strong positivity, and predominantly vesicular positivity, in the cytoplasm, especially malignant tumors, such as SCC, MGT, OM, and SM, and, thus, these tumors were expected to express more tumor-associated molecules. Even though no direct evidence of a connection between tumor development and legumain expression has been widely presented, we speculate that these characteristics may explain why legumain was overexpressed, especially in the epithelial tumors. However, further functional studies are highly recommended if the functions of legumain expression in canine carcinogenesis are to be uncovered.

The high levels of legumain expressed by tumor cells suggests that the inhibition of legumain is a potential strategy against tumor progression, based on its facilitation of tumor growth and its specific expression in tumors [5]. Legumain-specific targeting therapy, using nanoparticles, has been effective for breast cancer in a mouse model without significant toxicity [30]. The relatively limited expression of legumain in normal tissues [3,31] and its overexpression in tumor-associated endothelial cells [8,31] were also found in the current study. These findings indicate that legumain has the potential to serve as an ideal therapeutic target in tumor-bearing dogs, especially in dogs with epithelial-type tumors. Though we did not identify either the intensity or the percentage of legumain expression in any specific tumor type, we did find that non-mesenchymal tumors expressed an elevated level of legumain. However, it would be more prudent to verify these findings using a larger sample size to support our results. Taken together, the findings show that legumain was overexpressed in the canine tumor samples in this study, and further studies are needed to elucidate these findings with a more comprehensive investigation.

In the original Western blot analysis (Figure S1), there were two bands in the M5, KMec, CMT-1, and MPG cell lines, since, in UL-1 and CLBL-1 cell lines, this does not happen. Besides, there is a positive signal in the near 75kDa band. To our best knowledge, this is the first study to investigate legumain expression in canine tissues; we cannot find any clues why there are variations among the different cell lines. One of our possible speculations for the 75kDa band may be the different legumain isoforms. From the NCBI protein database, at least nine isoforms were recorded (while it seems only three complete isoform amino acids were shown with the corresponding molecular weight around 50 kDa). Therefore, we hypothesize the 75kDa band might be one of the isoforms while more data are needed to verify the hypothesis. However, for the antibody we used in this study, the immunogen is the partial amino acid sequence of human legumain. This sequence identity between human and canine species is 91% to prove this antibody could specifically react with canine legumain. 

In this study, we found increased levels of legumain in canine tumors; however, this study had some limitations. First, this was a preliminary investigation that involved screening ten types of canine neoplasms with a relatively small sample size (*n* = 5–20) per tumor, and further investigations that focus on certain types of tumors are needed to support our findings. Then, the isoform of the canine legumain should be elucidated with further investigation. Studies with more biological experiments, such as examining functional assays using the knock-down or overexpression of legumain in canine cancer cell lines, could possibly validate the roles and even the mechanisms of legumain in canine tumorigenesis. Overall, even though more studies are needed to address these issues, the current study is the first to demonstrate that legumain has a high potential to serve as a biomarker in dogs with cancers.

## 5. Conclusions

In conclusion, this study first identified the heightened levels of legumain in canine tumors, which suggested that legumain may also act as a tumor antigen in the promotion of tumor development. Additionally, the levels of legumain significantly increased in epithelial- and other non-mesenchymal-type tumors. The overexpression of legumain potentially facilitates tumor formation in dogs with cancer, indicating that legumain could be a potential tumor biomarker and/or therapeutic target, with more investigation. Though further studies are necessary to define the clinical value and the usefulness of legumain as a therapeutic candidate, the elevated patterns of legumain in canine tumors are reported, for the first time, in this study.

## Data Availability

The data presented in this study are available on request from the corresponding author.

## Supplementary Materials

The following supporting information can be downloaded at: https://www.mdpi.com/article/10.3390/ani12040504/s1, Figure S1: Original figures of western blot analysis.
