# Supplementary material for: The High Expression of Legumain in Canine Neoplasms: A Retrospective Analysis of 100 Cases
Source: Animals (Basel). 2022 Feb 17;12(4):504. doi: 10.3390/ani12040504 (PMC8868329; doi:10.3390/ani12040504)
Supplement: Supplementary file 1 [file animals-12-00504-s001.zip › animals-1527310-supplementary.pdf]

Origunal Blots

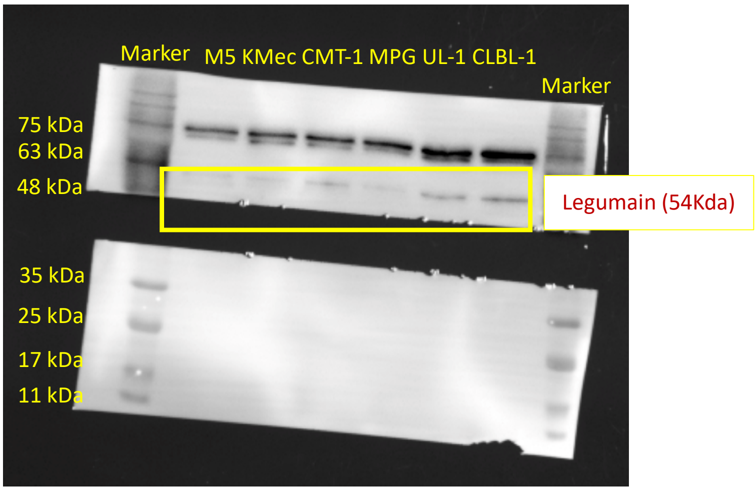

Figure S1. Western blot of the anti-legumain antibody in different canine cancer cell lines. The blot membrane was cut based on the standard band positions. We cropped this membrane at the 48kDa because we would like to test whether another antibody (about 37 kDa) could identify the correspondent proteins (Data were not shown in this study). (Antibody: anti-legumain at a 1:1,000 dilution; Exposure time: 1 second)

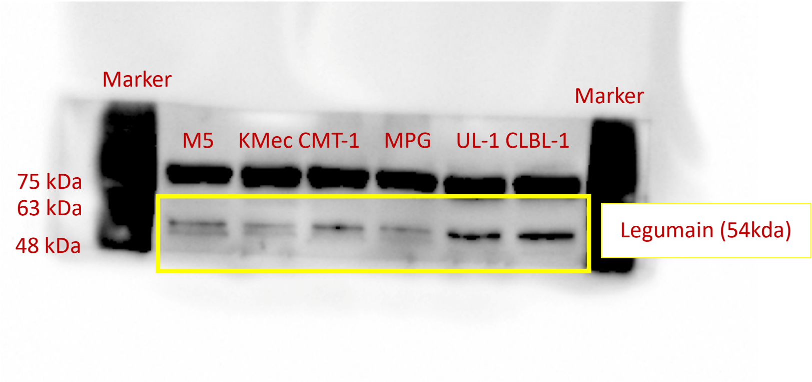

Figure S2. The original figure of the upper blot membrane of Fig. 1.
To enhance the contrast of the target bands (~54 kDa), we increase the exposure time to 20 seconds, but the markers were blurred. Therefore, the original figure that we had provided (Figure S1) is to combine the loading marker in Figure 1

and the protein bands in Figure 2.
(Antibody: anti-legumain at a 1:1,000 dilution; Exposure time: 20 seconds)

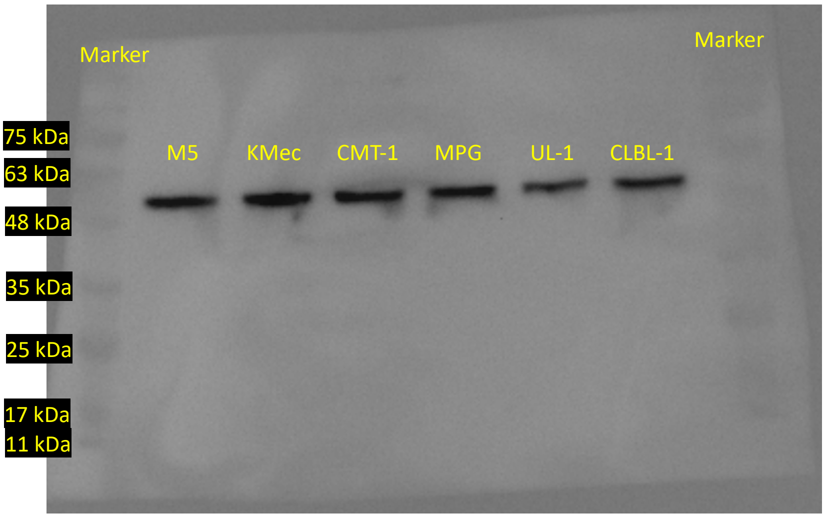

Figure S3. The uncropped figure of western blot analysis. Western blot of the anti-alpha tubulin antibody in different canine cancer cell lines.
(Antibody: anti-αtubulin at a 1:5,000 dilution, ~54 kDa)

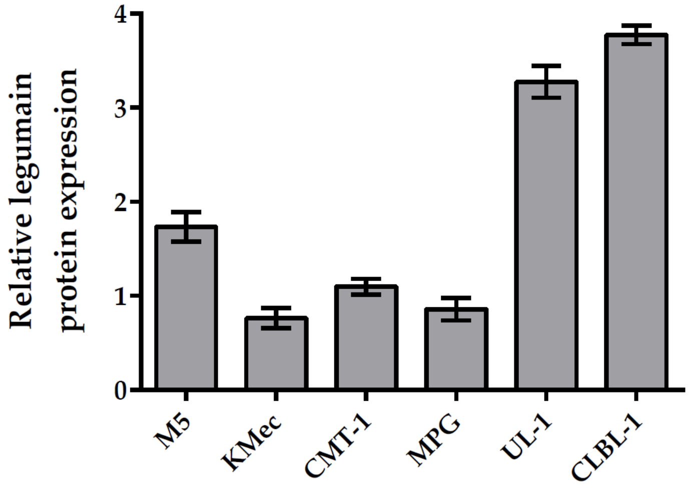

Figure S4. Relative legumain expression in different canine cell lines. The graph shows the relative legumain expression compared with α-tubulin (n=3).

The data were presented as mean ± SEM.
